# Comparison of Two Quantitative Real Time PCR Assays for *Rickettsia* Detection in Patients from Tunisia

**DOI:** 10.1371/journal.pntd.0003487

**Published:** 2015-02-23

**Authors:** Abir Znazen, Hanen Sellami, Emna Elleuch, Zouhour Hattab, Laroussi Ben Sassi, Fatma Khrouf, Hassen Dammak, Amel Letaief, Mounir Ben Jemaa, Adnene Hammami

**Affiliations:** 1 Laboratory of Microbiology, Research Laboratory “MPH”, Habib Bourguiba University Hospital of Sfax, Sfax University, Sfax, Tunisia; 2 Infectious diseases department, Hedi Chaker University Hospital of Sfax, Sfax University, Sfax, Tunisia; 3 Infectious diseases department, Farhat Hached University Hospital of Sousse, Sousse, Tunisia; 4 Medical department, Regional Hospital of Zarzis, Zarzis, Tunisia; 5 Laboratory of entomology, Pasteur Institute of Tunis, Tunis, Tunisia; 6 Intensive care unit, Habib Bourguiba University Hospital of Sfax, Sfax University, Sfax, Tunisia; University of Texas Medical Branch, UNITED STATES

## Abstract

**Background and objectives:**

Quantitative real time PCR (qPCR) offers rapid diagnosis of rickettsial infections. Thus, successful treatment could be initiated to avoid unfavorable outcome. Our aim was to compare two qPCR assays for *Rickettsia* detection and to evaluate their contribution in early diagnosis of rickettsial infection in Tunisian patients.

**Patients and methods:**

Included patients were hospitalized in different hospitals in Tunisia from 2007 to 2012. Serology was performed by microimmunofluorescence assay using *R. conorii* and *R. typhi* antigens. Two duplex qPCRs, previously reported, were performed on collected skin biopsies and whole blood samples. The first duplex amplified all *Rickettsia* species (PanRick) and *Rickettsia typhi* DNA (Rtt). The second duplex detected spotted fever group *Rickettsiae* (RC00338) and typhus group *Rickettsiae* DNA (Rp278).

**Results:**

Diagnosis of rickettsiosis was confirmed in 82 cases (57.7%). Among 44 skin biopsies obtained from patients with confirmed diagnosis, the first duplex was positive in 24 samples (54.5%), with three patients positive by Rtt qPCR. Using the second duplex, positivity was noted in 21 samples (47.7%), with two patients positive by Rp278 qPCR. Among79 whole blood samples obtained from patients with confirmed diagnosis, panRick qPCR was positive in 5 cases (6.3%) among which two were positive by Rtt qPCR. Using the second set of qPCRs, positivity was noted in four cases (5%) with one sample positive by Rp278 qPCR. Positivity rates of the two duplex qPCRs were significantly higher among patients presenting with negative first serum than those with already detectable antibodies.

**Conclusions:**

Using qPCR offers a rapid diagnosis. The PanRick qPCR showed a higher sensitivity. Our study showed that this qPCR could offer a prompt diagnosis at the early stage of the disease. However, its implementation in routine needs cost/effectiveness evaluation.

## Author Summary

Rickettsial diagnosis is challenging in routine laboratory. Serology offers only retrospective diagnosis. We aimed to introduce molecular methods in routine diagnosis of these infections. The lack of standardized methods led us to compare real time PCR assays previously reported in order to implement a clear strategies for diagnosis of these infections in our laboratory. Real time PCR proposed by Renvoisé *et al* includes two PCRs, one to detect spotted fever group and another to detect typhus group *Rickettsiae*. The real time PCR proposed by Giullieri *et al* includes a first PCR detecting 16rDNA of all *Rickettsiae* and if it is positive a second PCR detecting *R. typhi* should be performed. This second Real time PCR was shown to offer a slight higher sensitivity with a lower cost in our study. Skin biopsy specimens were more likely to show positive results than whole blood samples. Finally, positivity rates were higher among patients presenting at the first stage of the disease, essentially with negative serology.

## Introduction

Rickettsioses are vector borne diseases caused by Gram negative obligate intracellular rods belonging to the genus *Rickettsia* [1]. This genus contains 28 validated species, which are divided into four groups: typhus group (TG), spotted fever group (SFG), the ancestral group and the transitional group [2,3]. Typically, clinical features include eruptive fever associated or not with a unique or multiple inoculation eschar. However, spotless fever, absence or multiple inoculation eschars are frequently reported in endemic regions[4]. The diagnosis of Rickettsial infection remains a challenge because of these polymorphic and frequently non specific clinical presentations. In addition, due to its intracellular characteristics, the isolation of *Rickettsia* is difficult and limited to only reference laboratories. Thus, serology is the most widely used test in routine laboratory for the diagnosis of Rickettsial infections. However, serology has low sensitivity essentially in the early stage of the infection. Raoult *et al* reported that a combination of three serological methods had a sensitivity of only 56% to detect *R. africae* antibodies [5]. Besides, the mean delay of IgM seroconversion is 16 days in Mediterranean spotted fever and it could reach 25 days in African tick fever, for IgG seroconversion delays are 22 and 28 days respectively [6]. When patients received doxycycline, antibodies may not appear or appear late. Thus, late follow-up serological tests are needed and diagnosis is only retrospective. Since delayed diagnosis is a factor of poor prognosis, a rapid diagnostic method is needed. In an Algerian series, the authors reported a high prevalence of severe forms (49.1%) with 50% mortality rate[7]. The majority of these patients received ineffective antibiotic therapy before being hospitalized[7]. Thus, rapid methods for diagnosis, such as PCR, are necessary for successful treatment. Many PCRs were reported to be sensitive and specific to detect *Rickettsia* DNA either in skin biopsies or in whole blood of infected patients. Four genes were mainly used for molecular detection and diagnosis of *Rickettsia*: citrate synthase encoding gene (*gltA*)[8], genes for outer membrane proteins A and B (*ompA* and *ompB*) [9,10] and the 17kDa lipoprotein precursor antigen gene (*17 kDa*)[11]. To improve the sensitivity of conventional PCR, nested PCR was proposed, but this technique generates many contaminations and is no more recommended for diagnosis. Diagnosis of *Rickettsia* infection would benefit from the use of the more rapid and sensitive method of quantitative real time PCR (qPCR). In fact, qPCR was reported to be less expensive and reduces the delay of the diagnosis. Since the first report of the development of qPCR in the diagnosis of rickettsioses [12], many authors proposed several targets which were either species specific or detecting whole *Rickettsia* genus. Currently, molecular diagnosis of rickettsial diseases remains unstandardized with no available commercialized kits. Renvoisé *et al* reported a high sensitive qPCR for rickettsial diagnosis. The assay was used widely during 2 years in the French national reference centre and allowed to reduce the diagnosis delay [13]. Recently, Giulieri *et al*. proposed a q-PCR targeting 16srDNA showing high analytical sensitivity and specificity. However, this PCR assay was evaluated on a small number of samples[14].

In this study, we proposed to compare the two qPCRs for *Rickettsia* detection and to evaluate their contribution to the early diagnosis of rickettsial infection in Tunisian patients.

## Materials and Methods

### Sampling

**Skin biopsies**. Patients included in this study were hospitalized at different infectious disease departments from June 2007 to July 2012. They were suspected to have rickettsial infection on the basis of clinical presentation (acute fever with cutaneous rash) and epidemiologic feature (hot season, increased exposure to ticks and/ or fleas). Three Tunisian hospitals participated to the study. The Hedi Chaker University of Sfax is the unique tertiary care hospital in the south of Tunisia and patients enrolled are from different regions. Patients with eruptive fever hospitalized during 2011 at The Infectious Diseases Department Farhat Hached University Hospital of Sousse and at The Regional Hospital of Zarzis were also included. For each patient, a skin biopsy of the inoculation eschar or the cutaneous rash, a whole blood sample in EDTA and a serum sample were requested. The skin biopsies and whole blood samples were stored at -80°C until their use.

### Ethics statement

This study was approved by our institutional review board “Habib Bourguiba University hospital ethics committee” with the given number 11–13. All the subjects provided informed written consent (all our patients were adults and children were excluded).

### Serology

Serology was performed in a microimmunofluorescence assay using *R. conorii* and *R. typhi* antigens provided by the “Unité des Rickettsies, Marseille France” as described previously [15]. Titers equal to or higher than 1: 32 for IgM and 1: 128 for IgG were considered positive.

### DNA extraction

Total DNA from skin biopsies and 200 μl of whole blood were extracted using QIAamp DNA tissue extraction kit (Qiagen, Hilden, Germany) according to manufacturer’s instructions (protocols used were DNA purification from tissue and DNA purification from blood). Since all skin biopsies were performed using punch, weight of used tissues ranged between 18 and 20 mg. DNA was eluted in a final volume of 100μl. DNA extracts were stored at -20°C until their use.

### PCR amplification

Two duplex qPCR assays were used. The first set of primers and probes consisted of a qPCR targeting all *Rickettsiae* named PanRick and a qPCR targeting *R. typhi* named Rtt[14]. The second set consisted of two qPCR named RC0338 and Rp 278 detecting SFG and TG *Rickettsiae*, respectively[13]. QPCR amplifications and products detections were carried out in the CFX96 Touch Real-Time PCR Detection System (Biorad, USA). The reaction mixture included a final volume of 20 μL with 0.2 μM of each primer, 0.2 μM of probe and 12.5 μL of Premix ExTaq(Takara, Japan) and 5 μL of DNA sample. After a hot-start cycle at 95°C for 2 min, reactions were cycled 40 times as follows: 95°C for 15 s and 60°C for 1 min.

### Positive recombinant plasmid controls

*Rickettsia montanensis* DNA and *R. typhi* DNA (extracted from strains grown on cell culture and kindly provided by Prof Didier Raoult, Unité des Rickettsies, Marseille, France) was used as positive controls for SFG and TG *Rickettsia* detection respectively. To allow quantification, four plasmids containing the different targets were constructed. For each couple of primers, the genomic DNA was amplified using the polymerase Flexi (Promega, USA). PCR products were purified using Quick-PCR Purification Kit (Qiagen, Hilden, Germany) and then cloned into the pGEM-T Easy vector using the Kit (Promega, USA). The ligation mixtures were transferred into *E. coli* Top10 competent cells using the CaCl_2_ method. The presence of the appropriate insert was verified by PCR followed by sequencing. Thus, DNA products were analyzed on a standard 2% agarose gel containing ethidium bromide (Sigma). DNA sequences were elucidated by the dideoxynucleotide chain termination method according to a cycle sequencing protocol using thermosequenase (Amersham Pharmacia Biotech) with the DNA sequencer ABI PRISM 3100/3100-Avant Genetic Analyser. The identified positive colonies were grown in LB medium containing ampicillin (100 μg/ml), and the recombinant plasmids were isolated from bacteria cells using a QIAprep Spin Miniprep Kit (Qiagen, Hilden, Germany). Quantification was performed on a Nanodrop ND-1000 (Witech, Littau, Switzerland).

### Case definition

Patients were considered to have rickettsial infection when at least one of the following conditions was fulfilled; the occurrence of the characteristic triad of rickettsiosis (fever, eschar and cutaneous rush) during hot season (from May to October), or positive serology (positivity of both IgM and IgG on a single serum, seroconversion or significant elevation of IgG titers on two sera)

### Data quantification and analysis

To assess analytical sensitivity of the four qPCRs, seven serial 10-fold dilutions (from 10^6^ copies/μl to 1 copy/μl) of the four positive control plasmids were tested in triplicate. To determine the quantity of DNA in samples, five standards (10^4^, 10^3^, 10^2^, 10, 1 copies / μl) were tested in each run. Quantity of DNA was determined using the Bio-Rad CFX Manager software.

Frequencies were compared using the Student test (χ^2^) on EpiInfo software (version 6.0). Fisher exact test was used if numbers of subgroup were less than 5. Means were compared using t-test. For all used tests, the *p* value was considered significant when < 0.05.

## Results

**Fig 1:**
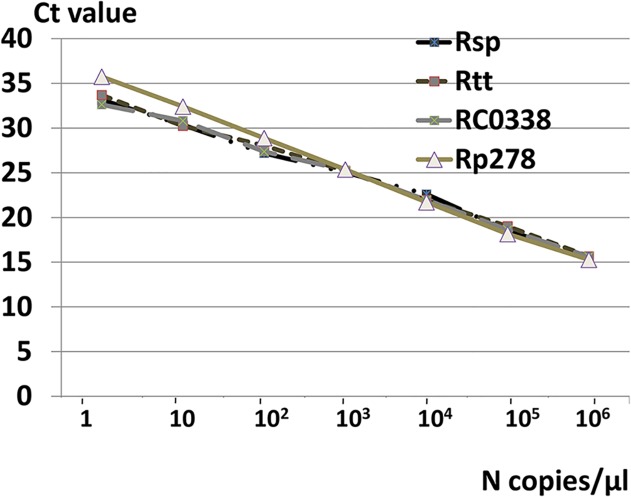
Standard curves constructed for the four quantitative PCRs.

To determine the sensitivities of the two duplex qPCRs, serial dilutions from 10^6^ to 1 copies/μl were produced for each target. All qPCRs were able to detect rickettsial DNA at a concentration of 2 copies/reaction. Constructed standard curves are shown in Fig. 1. All qPCRs showed good reproducibility when standards were tested in triplicates.

A total of 180 patients were included in the study. Serology was performed for all patients. Skin biopsy and whole blood samples were obtained from 77 and 174 patients respectively.

**Fig 2:**
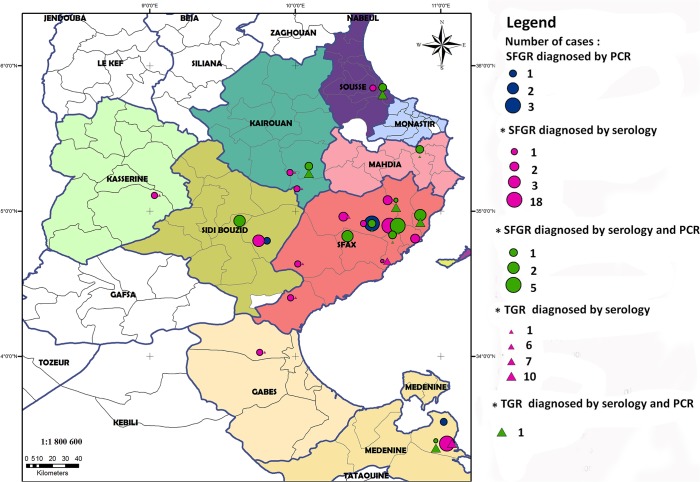
Geographic distribution of patients with rickettsioses hospitalized in the three medical centers (Sfax, Sousse and Zarzis). The size of circles and triangles is related to the number of patients diagnosed. SFGR: spotted fever group rickettsioses, TGR: typhus group rickettsioses.

Among the 180 tested patients, diagnosis of rickettsial infection was confirmed in 82 cases (45.5%). Serology was positive in 73 cases (89%) and qPCR in 46 cases (56%). The geographic distribution of patients with confirmed diagnosis using serological tests or molecular methods is shown in Fig. 2.

For skin biopsies PanRick qPCR was positive in 24 samples (54.5%) among 44 obtained from patients with confirmed diagnosis. When subjected to Rtt qPCR, three samples were positive. Using the second set of qPCRs, among 21 positive samples (47.7%), 19 were detected by RC0338 qPCR and 2 by Rp278 qPCR.

For whole blood samples, PanRick qPCR was positive in 5 cases (6.3%) among 79 specimens obtained from patients with confirmed diagnosis. When subjected to Rtt qPCR, two samples were positive. Using the second set of qPCRs, the positivity rate was of 5% with three samples positive by RC0338 qPCR and one sample positive by Rp278 qPCR.

Table 1 showed positivity rates in patients with negative serology at admission and patient with already detected antibodies at diagnosis. The positivity rates of qPCRs are higher in patients with negative serology at admission. Comparison of means of DNA quantities detected in skin biopsies obtained from patients with first positive serum versus patients with negative first serum did not show any statistical difference for the PanRick qPCR (p = 0.74) and RC00338 qPCR (p = 0.48). For the other qPCRs and whole blood samples, comparisons were not performed because of the limited numbers in each group.

**Table 1:** Positivity of qPCR according to serologic status among patients with confirmed diagnosis.

	Total	Serology (+) on first serum (N = 66)	Serology (-) on first serum (N = 16)	*p*
Skin biopsies	44	29	15	
PanRick	24(54.5%)	12 (41.4%)	12(80%)	**0.03**
Rtt	3(6.8%)	2(6.9%)	1(6.7%)	0.7
RC00338	19(43.2%)	10(34.5%)	9(60%)	0.1
Rp278	2(4.5%)	1 (3.4%)	1(6.7%)	0.5
Whole blood samples	79	63	16	
PanRick	5(6.3%)	2(3.1%)	3(18.7%)	**0.05**
Rtt	2(2.5%)	2(3.1%)	0	0.7
RC00338	3(3.8%)	0	3(18.7%)	**0.007**
Rp278	1(1.2%)	1(1.6%)	0	0.8

## Discussion

Our data confirmed that rickettsial infections, especially SFG rickettsioses, are endemic in our region. Diagnosis of these infections remains challenging, since laboratory conformation of rickettsial infection by serology (the most available method in routine use) is a retrospective process and could not be used to guide patient treatment. In addition, MIF assay requires well experienced technicians and lacks standardization. Alternatively, PCR was proposed. It permits a rapid diagnosis and could improve the outcome. First, many conventional PCRs targeting many genes (*gltA*, *ompB*, *ompA*) were used but their sensitivity is diminished. Recently, real time PCR, largely used in rickettsiology, is considered as a rapid and very sensitive molecular tool[16]. Initially, it was used to study the susceptibility of *Rickettsia* species to antibiotics since it allows quantification of DNA[17]. Currently, many studies proposed qPCRs to detect rickettsial DNA in clinical samples. Some of these qPCRs are species specific and others are genus specific allowing detection of a wide range of rickettsial pathogens [12,18,19]. Compared to conventional PCR, qPCR is more sensitive and is less time consuming, but the higher cost of this technique limits its wide use [20]. Of note, real time thermal cyclers cost is high. However, if the instrument is available, the qPCR reaction cost could be diminished lower than that of conventional PCR. In fact, many efficient and less expensive reagents, such as that used in our study (Takara), are currently available. Besides, conventional PCR uses ethidium bromide and UV light to visualize amplification products in the agarose gel medium while qPCR uses fluorescent dye system which is safer. Another limitation of qPCR is the high risk of contamination, but it remains lower than that of nested PCR[20]. In this study, we compared two previously reported duplex qPCRs to detect rickettsial DNA in clinical samples. The duplex qPCRs compared were able to detect all rickettsial DNA and to differentiate between SFG and TG using different approaches. The duplex developed by Giulieri *et al* [14] is composed of a first qPCR targeting the 16s rDNA gene that detect all *Rickettsia* species and a second qPCR detecting *R. typhi*, which is performed only if the first qPCR is positive. The duplex used by Renvoisé et al [13] consisted of two qPCRs that should be performed in parallel since the first qPCR detects SFG *Rickettsia* species and the second qPCR detects TG rickettsial DNA. The analysis of standard curves showed that the two duplex qPCRs had comparable sensitivities (up to two copies of DNA were detected). When applied to clinical samples, panRick qPCR was slightly more sensitive to detect rickettsial DNA essentially for SFG *Rickettsia*. When previously described, this qPCR was not evaluated on a large clinical sample. In our study, PanRick qPCR confirmed diagnosis of rickettsioses for 70.6% of patients with skin biopsies. Finally, the PanRick qPCR would be less expensive since the PCR targeting TG is performed only when the first qPCR is positive. To have identification at species level, more specific qPCRs or standard PCRs could be further performed.

Both qPCRs used showed higher rates of positivity when performed on skin biopsies than on whole blood. Previously, Renvoisé *et al* [13] found that among 45 positive clinical samples, 68.9% were skin biopsies and 4.4% were whole blood. In fact, a rickettsemia has been demonstrated to occur on the first stage of the disease. In our study, since institutions included were either tertiary care (Sfax and Sousse) or secondary care (Zarzis) hospitals, the majority of our patients had taken antibiotics before hospitalization with a delay exceeding frequently three days. Angelakis *et al* [21], showed in a recent study that the positivity is affected by the quantity of bacterium in the sample and that previous antibiotic treatment reduces the number of *Rickettsia spp*. in the skin biopsy. QPCRs used in our study showed higher positivity in patients with negative serology with a statistically significant difference. In fact, when antibodies are detectable, the number of bacteria is decreased both in blood and in the inoculation site.

TG *Rickettsia* species detected in our study has been previously reported in our country[22]. However, all reported cases were diagnosed using serology. In this report we confirmed diagnosis of murine typhus using molecular methods. Previously, we reported 43 cases of murine typhus diagnosed using MIF assay and we speculated that the disease is still endemic in our country[23]. Using qPCR, TG rickettsial DNA was detected both in whole blood samples and in skin biopsies taken from patients with eruptive fever, confirming murine typhus onset in our country. Effectively, Giulieri *et al* [14] reported in their study that the patient diagnosed with TG rickettsioses had traveled to Tunisia. Murine typhus was reported also in two French travelers from Tunisia [24].

In our study, many cases occurred during the summer of 2011 in the western south of Tunisia (Zarzis) and they were generally severe. In fact, in this region many refugees from Libya were living during this period and many of them had brought their animals. Unfortunately, no collected arthropods were obtained from these animals. It should be noted that we previously reported severe form of Israeli spotted fever rickettsioses in a patient suspected to be infected in Libya [25]. Thus, the circulation of virulent strains has to be confirmed by larger studies comparing strains in Southern East of Tunisia and Libya essentially.

In conclusion, qPCR is a very sensitive tool. The main advantage of the technique is that it offers a rapid diagnosis. Optimal use of the qPCR includes its application in patients with clinical features and epidemiological characteristics compatible with rickettsioses. Of note, the assays used in our study are not sensitive enough to allow ruling out diagnosis if negative results are obtained. However, diagnosis is more effective in patients with negative serology. Thus, the assay could be proposed as an alternative method for laboratories with limited budgets. Other qPCRs, essentially multiplex qPCRs, using most frequent species specific probes could be developed so as the diagnosis of rickettsial infections could be made more easily.

## Supporting Information

S1 Checklist STARD Checklist. (DOC)
